# Coated Pills

**Published:** 1882-08

**Authors:** 


### Coated Pills.

Of all the different methods of making pills tasteless by means of coating, the sugar and gelatine coating has been found to be the best. It has been said that pills must be dried out to allow a coating of sugar, and that is partly so, but drying out is necessary to keep the pills from spoiling and moulding. Suppose pills are coated while soft they *must be dried out after coating* or else they will spoil and cannot be kept in stock. The case stands so: If the coating of gelatine has to be done by means of needles, pills cannot be coated if they are hard, and therefore *must be coated while*

*soft*, but must be dried out after coating, while pills to be coated with sugar can be dried out *before* coating and thus do not need drying out afterwards. If the pills would be kept in stock without having been dried out they would become mouldly and would spoil. We can have pills which remain soft and do not become mouldy, but such pills have been made by adding grease to the pill-mass. If the proper excipient has been used, hard dried-out pills may dissolve quicker than soft ones. To prepare a pill-mass properly for making pills in large quantities, without resorting to means which should not be used, a long experience is required. Of such pills to be found in trade, which have been properly prepared according to theory and practical experience, we should mention the pills prepared by W. R. Warner & Co., of Philadelphia. These pills are soluble, can be kept in stock without spoiling and are always of the same nice appearance and uniformity.—*Apotheker Zeitung*, New York, March 15, 1882.

